# Single-Session Transcranial Direct Current Stimulation Temporarily Improves Symptoms, Mood, and Self-Regulatory Control in Bulimia Nervosa: A Randomised Controlled Trial

**DOI:** 10.1371/journal.pone.0167606

**Published:** 2017-01-25

**Authors:** Maria Kekic, Jessica McClelland, Savani Bartholdy, Elena Boysen, Peter Musiat, Bethan Dalton, Meyzi Tiza, Anthony S. David, Iain C. Campbell, Ulrike Schmidt

**Affiliations:** 1 Section of Eating Disorders, Department of Psychological Medicine, Institute of Psychiatry, Psychology & Neuroscience, King’s College London, London, United Kingdom; 2 Department of Psychosis Studies, Institute of Psychiatry, Psychology & Neuroscience, King’s College London, London, United Kingdom; Hospital Universitari de Bellvitge, SPAIN

## Abstract

**Background:**

Evidence suggests that pathological eating behaviours in bulimia nervosa (BN) are underpinned by alterations in reward processing and self-regulatory control, and by functional changes in neurocircuitry encompassing the dorsolateral prefrontal cortex (DLPFC). Manipulation of this region with transcranial direct current stimulation (tDCS) may therefore alleviate symptoms of the disorder.

**Objective:**

This double-blind sham-controlled proof-of-principle trial investigated the effects of bilateral tDCS over the DLPFC in adults with BN.

**Methods:**

Thirty-nine participants (two males) received three sessions of tDCS in a randomised and counterbalanced order: anode right/cathode left (AR/CL), anode left/cathode right (AL/CR), and sham. A battery of psychological/neurocognitive measures was completed before and after each session and the frequency of bulimic behaviours during the following 24-hours was recorded.

**Results:**

AR/CL tDCS reduced eating disorder cognitions (indexed by the Mizes Eating Disorder Cognitions Questionnaire-Revised) when compared to AL/CR and sham tDCS. Both active conditions suppressed the self-reported urge to binge-eat and increased self-regulatory control during a temporal discounting task. Compared to sham stimulation, mood (assessed with the Profile of Mood States) improved after AR/CL but not AL/CR tDCS. Lastly, the three tDCS sessions had comparable effects on the wanting/liking of food and on bulimic behaviours during the 24 hours post-stimulation.

**Conclusions:**

These data suggest that single-session tDCS transiently improves symptoms of BN. They also help to elucidate possible mechanisms of action and highlight the importance of selecting the optimal electrode montage. Multi-session trials are needed to determine whether tDCS has potential for development as a treatment for adult BN.

## 1. Introduction

Bulimia nervosa (BN) is characterised by recurrent episodes of binge-eating and inappropriate compensatory behaviours. It typically emerges during adolescence and is associated with substantial functional impairment, suicidality [1], and an increased risk of mortality [2, 3]. Furthermore, BN has high rates of comorbidity with major mood, anxiety, impulse control, and substance use disorders [4]. Lifetime prevalence estimates for young women are 7% when subthreshold cases are considered [1]. Cognitive behavioural therapy is regarded as the gold-standard treatment [5], yet most patients remain symptomatic following therapy [6] and attrition rates are as high as 50% [7].

Development of novel therapies for BN relies on identifying factors that contribute to pathogenesis. Evidence indicates that alterations in reward processing may play a central role; for example, patients with BN rate pictures of food as more interesting/arousing than healthy controls [8] and bulimic symptoms correlate positively with reward sensitivity [9, 10]. Neuroimaging data support the importance of reward systems in BN: both hyper- and hypo-responsivity have been observed in the neural networks that subserve anticipatory (wanting) and consummatory (liking) food reward processing [11–13]. Individuals with BN also appear to have deficient self-regulatory control, thus increasing instability and erratic responding to rewarding stimuli [13]. For example, BN [14] and binge-eating more generally [15] are associated with impaired reactive response inhibition, and our group recently observed an increased propensity to devalue delayed rewards (a concept known as temporal discounting; TD) in patients with BN relative to healthy controls [16, 17]. Neuroimaging studies suggest these difficulties are related to hypoactivity in circuitry that supports self-regulatory capacities [18, 19]. It has therefore been proposed that disturbed eating in BN is underpinned by problems in reward processing and self-regulatory control, which correspond to aberrations within ventral limbic and dorsal cognitive frontostriatal neural networks, respectively [12, 13, 20]. Negative mood may trigger binge-eating by altering the reward value of food [21, 22] and by diminishing self-regulatory processes [23].

Non-invasive brain stimulation (NIBS) enables targeted manipulation of cortical excitability, and may be useful for ‘normalising’ altered neural circuit activity in BN. The most common NIBS modalities are repetitive transcranial magnetic stimulation (rTMS) and transcranial direct current stimulation (tDCS). rTMS uses a coil to generate a magnetic field, which penetrates the skull and induces an electrical current, whereas tDCS delivers a low-amplitude direct current via two surface electrodes (anode and cathode). Although both methods are well-tolerated and have minimal side effects, tDCS has several practical advantages over rTMS: it is portable, inexpensive, has a more favourable safety-feasibility profile, and can be applied bilaterally.

Evidence for the usefulness of NIBS in psychiatry is accumulating, and the dorsolateral prefrontal cortex (DLPFC) has been the targeted site in most studies. This region is part of the dorsal cognitive frontostriatal circuitry—representing the major neural structure involved in executive functions, including self-regulatory control [24]–and is also implicated in reward processing due to its anatomical/functional connections with ventral limbic circuitry [25]. Given the aetiological relevance of these neurocognitive capacities in BN, manipulating the DLPFC with NIBS might alleviate symptoms of the disorder [26]. Indeed, our group found that one session of real versus sham rTMS over the left DLPFC was associated with a decreased urge to eat and fewer binge-eating episodes during the 24-hour follow-up period in 38 participants with a bulimic disorder [27], and Hausmann et al. [28] observed complete remission of binge/purge symptoms following 10 sessions of left DLPFC rTMS in a patient with refractory BN. Modulation of the DLPFC with tDCS has produced therapeutic effects in food cravers [29], obese individuals [30], and patients with various psychiatric disorders [31] (including anorexia nervosa and binge-eating disorder) [32, 33]; however, its utility in BN has not been explored.

This proof-of-principle clinical trial investigated the effects of two single sessions of sham-controlled tDCS administered bilaterally over the DLPFC (anode right/cathode left [AR/CL] and anode left/cathode right [AL/CR]) in patients with BN. The aims were to establish whether these sessions would temporarily: (i) suppress core symptoms of BN (urge to binge-eat, eating disorder [ED]-related cognitions, frequency of binge-eating and compensatory behaviours); (ii) reduce TD behaviour (an indicator of poor self-regulatory control); (iii) alter the wanting/liking of high- and low-calorie sweet and savoury foods (anticipatory/consummatory reward processing); and (iv) improve mood.

## 2. Materials and Methods

### 2.1. Participants

**Fig 1:**
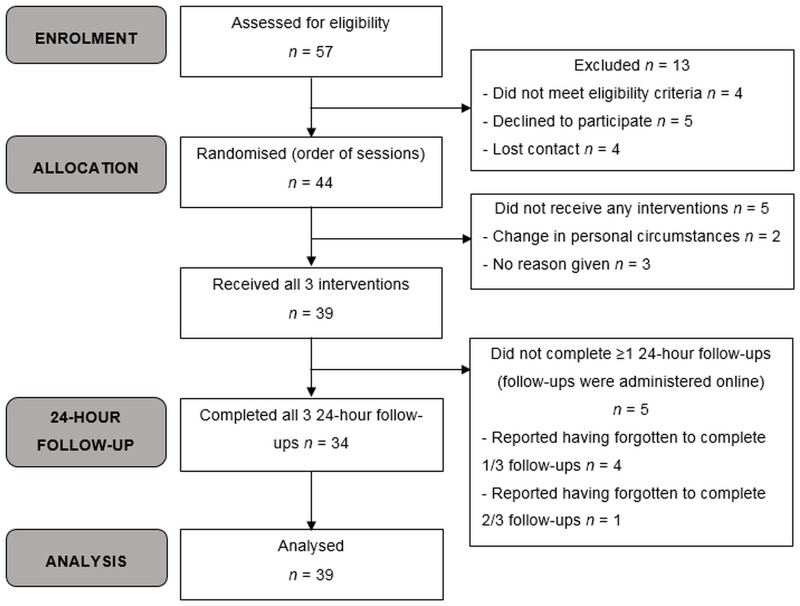
Consolidated Standards of Reporting Trials flow diagram of the progress through the phases of this randomised controlled trial.

Male and female volunteers (≥ 18 years) with BN were recruited from the King’s College London, Beat, Call for Participants, and Experimatch websites, and from the South London and Maudsley NHS Foundation Trust ED outpatient service. Respondents were screened by phone and a DSM-5 diagnosis of BN was confirmed with an adapted version of the Eating Disorder Diagnostic Scale (EDDS) [34]. Exclusion criteria were: (i) contraindications to tDCS [35] (details available on request); (ii) significant health problems in the previous six months; and (iii) pregnancy. Fifty-seven people (4 males) completed the telephone screen and 53 fulfilled the eligibility criteria (Fig 1). Of these, 39 (2 males) completed all 3 study sessions (the dropout rate was 0%) and 35 (2 males) completed all 3 follow-up questionnaires.

A two-way 2 x 3 repeated measures ANOVA sample size calculation (incorporating interaction effects) was conducted using G*Power, with a two-sided significance of 0.05 and a power of 0.95 (number of groups = 3; number of measurements = 6). This indicated that 36 participants were needed to detect a medium effect size (*f* = 0.25). Accounting for a 5% dropout rate, a total of 38 participants were required.

The study was conducted at the Institute of Psychiatry, Psychology & Neuroscience (London, UK). Ethical approval was obtained from the London City Road & Hampstead National Research Ethics Service committee (10^th^ February 2014, 14/LO/0025). All participants provided written informed consent and were debriefed at the end of the experiment. £50 was given to each participant as compensation for their time. The trial was registered at www.controlled-trials.com (29^th^ April 2014, ISRCTN70396934). Participants were recruited between 1^st^ May 2014 and 17^th^ August 2015, and data were collected between 20^th^ May 2014 and 9^th^ September 2015. The full trial protocol and supporting CONSORT checklist are available as supporting information (S1 and S2 Appendices).

### 2.2. Design and procedure

A double-blind sham-controlled crossover design was employed in which all participants received three sessions of tDCS: (i) AR/CL; (ii) AL/CR; and (iii) sham. In an effort to minimise any potential learning effects, order of stimulation was randomised and counterbalanced across participants by a third party using the block method (block size: 6). Electrode polarity for sham sessions was determined with a random number generator (0 or 1). Due to the inclusion of two electrode montages, it was not possible for the tDCS technician to be blinded; however, the patient and the researcher administering the experimental measures remained blind throughout. An intersession interval (≥ 2 days; *M* = 9.10, *SD* = 9.39) was used to avoid any carryover effects of stimulation and, for each participant, all three sessions were held at the same time of day.

**Fig 2:**
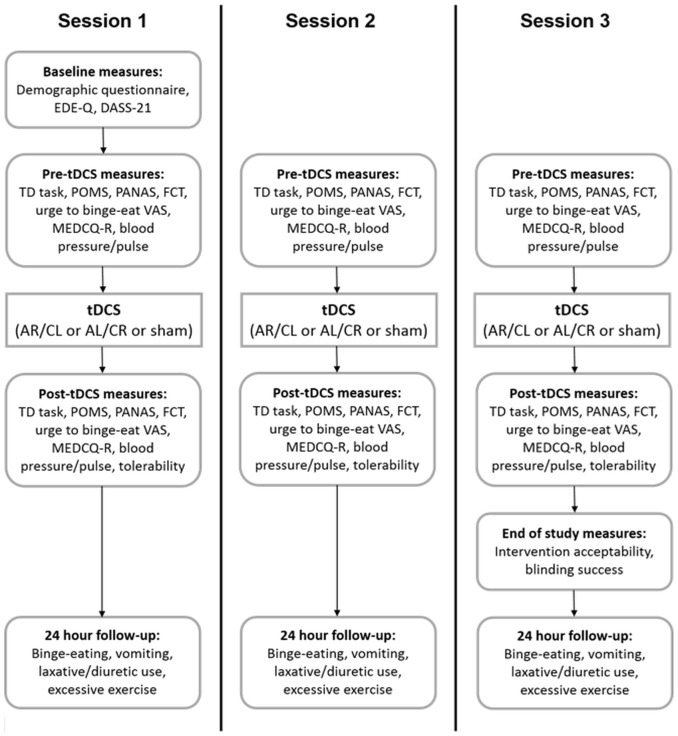
Schematic representation of study procedure.

At the first appointment, participants completed several baseline assessments (demographic questionnaire, Eating Disorder Examination Questionnaire [EDE-Q] [36], Depression Anxiety and Stress Scales [DASS-21] [37]). The following pre-tDCS measures were completed during each study session: (i) TD task; (ii) Profile of Mood States (POMS) [38]; (iii) Positive and Negative Affect Schedule (PANAS) [39]; (iv) Food Challenge Task (FCT); (v) urge to binge-eat visual analogue scale (VAS); (vi) Mizes Eating Disorder Cognition Questionnaire-Revised (MEDCQ-R) [40]; and (vi) blood pressure/pulse. Participants then received a 20-minute session of tDCS (AR/CL, AL/CR, or sham). Immediately post-tDCS, they repeated the pre-tDCS measures in the same order, followed by a VAS measuring the tolerability of tDCS. A follow-up questionnaire was completed 24 hours later. At the end of the third appointment, intervention acceptability and blinding success were evaluated. A schematic representation of the study procedure is provided in Fig 2.

### 2.3. Transcranial direct current stimulation (tDCS)

tDCS was delivered using a neuroConn^®^ DC-STIMULATOR (20 mins, 2 mA, 10-second ramp on/off) via two 25cm^2^ surface sponge electrodes soaked in 0.9% sodium chloride. In the AR/CL condition, the anode and cathode were placed over the right (F4) and left DLPFC (F3), respectively. This montage was reversed for AL/CR tDCS. The sites of stimulation were located using the Beam F3 Location System [41], which is based on the International 10–20 system. For sham tDCS, electrode placement corresponded to one of the active conditions (see Design and procedure). To mimic real stimulation, the device’s sham setting was used: a current was applied for the first 30 seconds of the session, after which it stopped automatically. Participants therefore experienced the initial tingling sensation but received no stimulation for the remaining 19.5 minutes. Our group has shown that this sham treatment cannot be distinguished from real tDCS [29].

### 2.4. Measures

Measures are described in the order in which they were administered. The primary outcome variable was urge to binge-eat; all other outcomes were secondary.

#### 2.4.1. Urge to binge-eat visual analogue scale (VAS)

Participants rated their urge to binge-eat on a computerised VAS administered via Adaptive Visual Analog Scales (AVAS) software [42], which was anchored with “no urge to binge-eat” and “extreme urge to binge-eat”.

#### 2.4.2. Mizes eating disorder cognition questionnaire-revised (MEDCQ-R)

The MEDCQ-R (formerly the Mizes Anorectic Cognitions Questionnaire-Revised) [40] is a 24-item self-report inventory which assesses cognitions in EDs. Responses are made on a Likert scale ranging from 1 (strongly disagree) to 5 (strongly agree), yielding a global score and a score for three subscales: self-control and self-esteem, rigid weight regulation and fear of weight gain, and weight and approval. Interpretation of the scores involves converting raw scores into T-scores using the following formula: (obtained raw score—mean)/standard deviation x 10 + 50. Appropriate normative data are published elsewhere [43]. Global MEDCQ-R T-score was used as the outcome variable for this measure.

#### 2.4.3. Food challenge task (FCT)

The FCT—which involves exposure to a food video, the presentation of real food, and a series of VASs—was initially developed by our group to induce and assess food cravings, and has been administered in our laboratory in variable formats [27, 29, 44–46]. Based on recent literature [47] and on feedback from participants in earlier studies [29, 46], several adaptations were made to the FCT for the present trial. Firstly, a new food video was created (5 mins) using clips from television advertisements. This video was piloted in 40 adults (9 males), who rated the foods shown as highly appetising (mean rating: 73.38/100) and whose hunger was significantly increased by the footage [*t*(39) = -6.37, *p* < .001, *r* = 0.71]. Secondly, the real foods presented were altered to cover four categories: high-calorie sweet (chocolate, sweets), high-calorie savoury (crisps, nuts), low-calorie sweet (orange, apple), and low-calorie savoury (table water crackers, rice cakes). Lastly, the VASs—which were computerised and administered via AVAS—were modified so that both the wanting (craving) and the perceived liking of each real food were measured. For wanting, participants were asked “How much do you want some of the [food] right now?” and, for liking, they were asked “How pleasant would it be to experience the taste of the [food]?”. These questions have been used previously to differentiate the wanting and liking elements of food reward [47].

#### 2.4.4. Temporal discounting (TD) task

TD was assessed with a hypothetical monetary choice task, modelled on an established paradigm [48, 49]. Eighty binary choices were administered in a random order: for each one participants chose between a smaller amount of money available immediately (smaller-sooner [SS] reward) and a larger amount available in 3 months (larger-later [LL] reward). Two types of decision framing were employed: Accelerate and Delay (40 binary choices in each set). In the Accelerate set, the LL reward remained at £100 and the SS reward increased from £20 to £98 in £2 increments. In the Delay set, the SS reward was fixed at £50 while the LL reward increased from £52 to £130 in £2 increments. TD was quantified by determining participants’ discount factor—the magnitude of reduction in the present value of a future reward—for each choice set using a two-step procedure described elsewhere [48–50] (the value obtained ranges from 0 to 1, with smaller numbers indicating greater TD and thus a greater tendency to choose the immediate reward). The global discount factor was calculated as the mean of the Accelerate and Delay discount factors, and used as the outcome variable.

#### 2.4.5. Profile of mood states (POMS)

The POMS [38] is a self-report measure containing 65 adjectives which respondents rate on a Likert scale ranging from 0 (not at all) to 4 (extremely). Participants answered in relation to how they were feeling at the time of responding (“right now”). The scale includes six factors (tension, depression, anger, vigour, fatigue, and confusion). A total mood disturbance score can also be calculated (global POMS score), which was used as the outcome variable for this questionnaire.

#### 2.4.6. Positive and negative affect schedule (PANAS)

The PANAS [39] consists of two 10-item self-report scales which measure positive and negative affect. On a Likert scale ranging from 0 (very slightly or not at all) to 4 (extremely), participants rate the extent to which they have experienced each of the 20 descriptors within a particular time frame (“right now” in the current study). Two scores are generated: positive (PANAS-positive) and negative (PANAS-negative) affect.

#### 2.4.7. Tolerability, acceptability, and blinding of tDCS

Tolerability was assessed with a 10cm paper-based VAS measuring level of discomfort experienced during the tDCS. Acceptability was determined by asking participants whether they would consider taking part in a therapeutic trial of tDCS (involving ~20 sessions), if it were available. The validity of the sham treatment was judged by asking participants and researchers who administered the experimental measures to identify the placebo session, and to rate their confidence in their answer on a 10cm paper-based VAS.

#### 2.4.8. Follow-up questionnaire

The follow-up questionnaire was administered online (the URL was shared by email). Participants were required to state how many episodes of binge-eating, vomiting, laxative/diuretic use, and excessive exercise they had engaged in during the 24-hour period following each tDCS session.

### 2.5. Data analysis

Statistical analyses were performed on IBM^®^ SPSS^®^ (version 21) using a two-sided significance of 0.05. A series of boxplots indicated that there were no obvious outliers in the data. For variables with normally distributed data, effects of tDCS were evaluated using two-way 3 (stimulation: AR/CL vs. AL/CR vs. sham) x 2 (timepoint: pre-tDCS vs. post-tDCS) repeated measures ANOVAs, whereby significant stimulation x timepoint interactions indicated that the effects of stimulation varied across conditions (simple effects analyses were used to determine which conditions differed). Where data were not normally distributed, Wilcoxon signed-rank tests were used to compare pre- and post-tDCS scores for each condition separately. Friedman’s one-way ANOVAs and one-way repeated measures ANOVAs were used to explore the effect of stimulation type (AR/CL vs. AL/CR vs. sham) on symptoms during the 24-hour follow-up period and on the discomfort experienced during tDCS, respectively. Blinding success was appraised using Pearson’s chi-square goodness-of-fit tests. Where relevant, effect sizes (*r*) are reported.

## 3. Results

### 3.1. Demographic and clinical characteristics

The sample comprised 37 females and 2 males aged 18–48 (*M* = 25.85, *SD* = 6.62) with a mean BMI of 21.65 (*SD* = 3.20). The majority were right-handed (87.2%), described their ethnicity as “white” (74.4%), and had an annual personal income < £20,000 (61.5%). All participants were educated to A Level standard or higher (a qualification offered to students completing secondary or pre-university education in the UK). The mean global EDE-Q score was 4.21 (*SD* = 1.06)—with 61.5% of scores above the clinically relevant cut-off [≥ 4; [51])—and severe or extremely severe levels of depression (≥ 21), anxiety (≥ 15), and stress (≥ 26; indexed by the DASS-21) were reported by 56.4%, 43.6%, and 35.9% of participants, respectively. Further information on clinical characteristics is provided in Table 1.

**Table 1:** Clinical characteristics of the study sample.

	M (*n*)	SD (%)	Range
**Duration of illness (months)**	110.87	95.62	4.00–528.00
**Time spent in treatment (months)**	22.97	42.29	0.00–183.60
**Current treatment**			
Psychotherapy	(13)	(33.3)	-
Pharmacotherapy	(4)	(10.3)	-
None	(22)	(56.4)	-
**Been an inpatient?**			
Yes	(10)	(25.6)	-
No	(29)	(74.4)	-
**Bulimic behaviours per week**^a^			
Binge-eating	8.08	13.80	1.00–87.50
Self-induced vomiting	8.10	14.25	0.00–88.00
Laxative/diuretic abuse	1.15	3.06	0.00–14.00
Fasting	2.44	2.44	0.00–10.00
Excessive exercise	2.16	2.57	0.00–10.00
**EDE-Q**^b^			
Restraint	3.92	1.21	0.60–6.00
Eating concern	3.86	1.14	1.40–6.00
Shape concern	4.68	1.24	0.75–6.00
Weight concern	4.38	1.33	1.40–6.00
Global	4.21	1.06	1.65–6.00
**DASS-21**^c^			
Depression	20.62	10.39	0.00–38.00
Anxiety	15.23	11.65	0.00–42.00
Stress	21.97	10.19	2.00–42.00

M, mean; SD, standard deviation; EDE-Q, Eating Disorder Examination Questionnaire; DASS-21, Depression Anxiety and Stress Scales.

^a^ Self-reported during the telephone screen.

^b^ Subscale and global scores can range from 0–6.

^c^ Subscale scores can range from 0–42.

### 3.2. Effects of transcranial direct current stimulation (tDCS)

#### 3.2.1. Eating disorder (ED) symptoms

**Fig 3:**
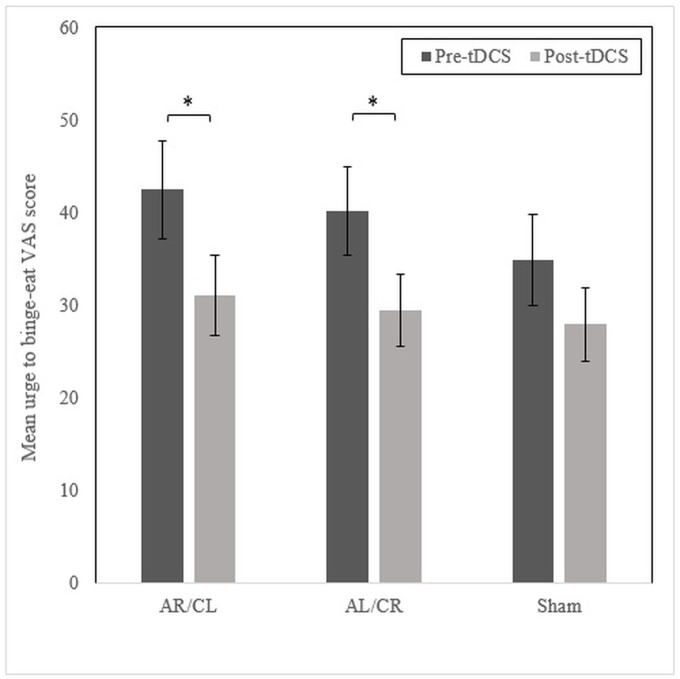
Mean urge to binge-eat VAS scores pre and post AR/CL, AL/CR, and sham tDCS. VAS, visual analogue scale; AR/CL, anode right/cathode left; AL/CR, anode left/cathode right; tDCS, transcranial direct current stimulation. * *p* < .05. Note: pre-tDCS scores across the three conditions were not significantly different [*χ*^2^(2) = 5.59, *p* = .061].

**Fig 4:**
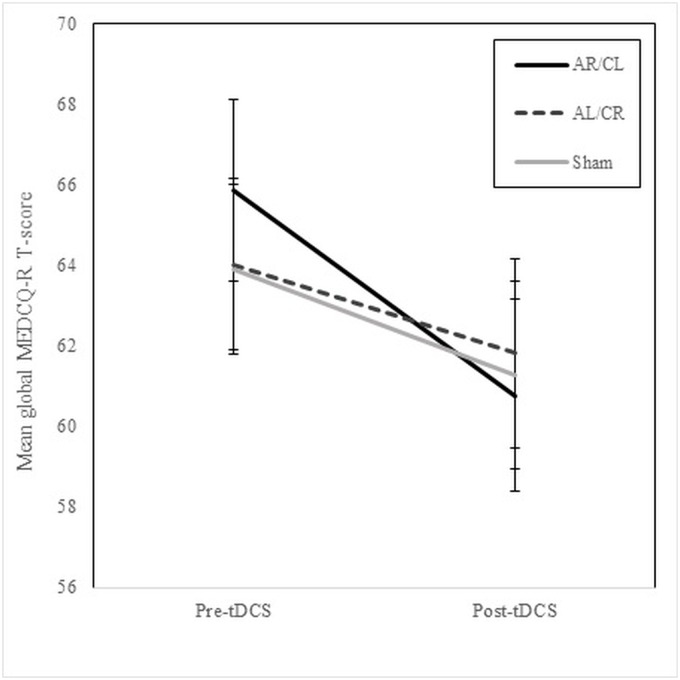
Mean global MEDCQ-R T-scores pre and post AR/CL, AL/CR, and sham tDCS. MEDCQ-R, Mize’s Eating Disorder Cognition Questionnaire-Revised; AR/CL, anode right/cathode left; AL/CR, anode left/cathode right; tDCS, transcranial direct current stimulation. Note: pre-tDCS scores across the three conditions were not significantly different [*F*(2, 76) = 2.05, *p* = .136].

Three Wilcoxon signed-rank tests demonstrated that urge to binge-eat VAS scores were reduced following active [AR/CL: Z = -2.42, *p* = .016, *r* = -0.27, AL/CR: Z = -2.52, *p* = .012, *r* = -0.28] but not sham stimulation [Z = -1.26, *p* = .207, *r* = -0.14] (Fig 3). For global MEDCQ-R T-score, a repeated measures ANOVA revealed a significant main effect of timepoint [*F*(1, 38) = 11.92, *p* = .001], but not stimulation [*F*(2, 76) = 0.30, *p* = .744], and a significant stimulation x timepoint interaction (with a Huynh-Feldt correction; *ε* = .82) [*F*(1.63, 62.02) = 3.83, *p* = .035] (Fig 4). Simple effects analyses showed that AR/CL stimulation reduced global MEDCQ-R T-scores significantly more than both AL/CR [*F*(1, 38) = 4.42, *p* = .042, *r* = 0.32] and sham stimulation [*F*(1, 38) = 5.17, *p* = .029, *r* = 0.35], and that AL/CR and sham tDCS exerted equivalent effects [*F*(1, 38) = 0.22, *p* = .643, *r* = 0.08]. Several Friedman’s ANOVAs showed that the frequency of binge-eating, vomiting, laxative/diuretic use, and excessive exercise during the 24-hour follow-up period was comparable across the three conditions [all *p* ≥ .549].

#### 3.2.2. Wanting and liking of food

Repeated measures ANOVAs were conducted to assess the effects of tDCS on the wanting and liking of each food separately, all foods together, sweet foods, savoury foods, high-calorie foods, low-calorie foods, sweet high-calorie foods, sweet low-calorie foods, savoury high-calorie foods, and savoury low-calorie foods. There was a significant main effect of timepoint across all liking [all *p* ≤ .020] but no wanting variables [all *p* ≥ .091]. Non-significant main effects of stimulation [all *p* ≥ .123] and stimulation x timepoint interactions were observed for all wanting/liking outcomes [all *p* ≥ .100].

#### 3.2.3. Temporal discounting (TD) behaviour

**Fig 5:**
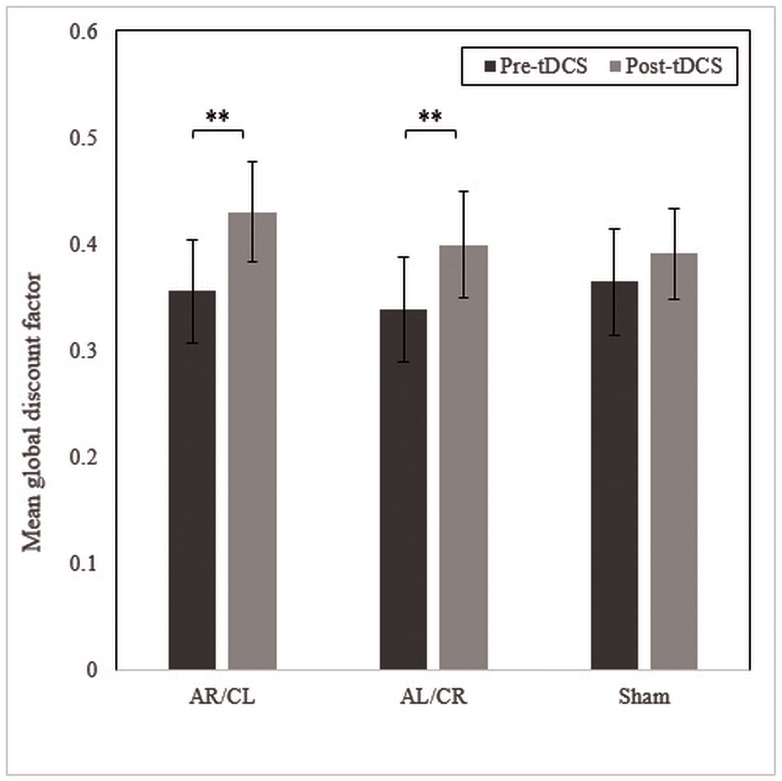
Mean global discount factors pre and post AR/CL, AL/CR, and sham tDCS. AR/CL, anode right/cathode left; AL/CR, anode left/cathode right; tDCS, transcranial direct current stimulation. ** *p* < .01. Note: pre-tDCS scores across the three conditions were not significantly different [*χ*^2^(2) = 1.61, *p* = .446].

Wilcoxon signed-rank tests showed that post-tDCS global discount factors were significantly higher (indicating increased self-regulatory control) than pre-tDCS scores following AR/CL [Z = -2.91, *p* = .004, *r* = -0.33] and AL/CR [Z = -3.04, *p* = .002, *r* = -0.34] tDCS, but not sham tDCS [Z = -1.74, *p* = .083, *r* = -0.20] (Fig 5).

#### 3.2.4. Mood

A repeated measures ANOVA showed a significant main effect of timepoint [*F*(1, 35) = 21.73, *p* = < .001], no main effect of stimulation [*F*(1.47, 51.44) = 2.19, *p* = .135], and a trend towards a significant stimulation x timepoint interaction for global POMS score [*F*(2, 70) = 2.92, *p* = .060]. Simple effects analyses demonstrated that AR/CL tDCS lowered global POMS scores significantly more than sham stimulation [*F*(1, 35) = 5.15, *p* = .030, *r* = 0.36]. In addition, although the two active conditions exerted similar effects on global POMS scores [*F*(1, 35) = 0.82, *p* = .371, *r* = 0.15], AL/CR tDCS was not significantly superior to sham stimulation [*F*(1, 35) = 2.32, *p* = .137, *r* = 0.25]. Two further repeated measures ANOVAs revealed a significant main effect of timepoint for PANAS-negative [*F*(1, 38) = 16.72, *p* < .001] but not PANAS-positive [*F*(1, 38) = 0.62, *p* = .435] score, and non-significant main effects of stimulation [all *p* ≥ .395] and interaction terms [all *p* ≥ .516] for both variables.

### 3.3. Success of the blinding procedure

Neither participants [41.0% correct; *χ*^2^(1) = 1.04, *p* = .308] nor researchers who administered the experimental measures [40.5% correct; *χ*^2^(1) = 0.87, *p* = .352] distinguished real from sham tDCS at a rate better than chance. Both parties expressed little confidence in their identification of the placebo session (10cm VAS, participants: *M* = 3.18, *SD* = 2.37, researchers: *M* = 0.58, *SD* = 1.44).

### 3.4. Safety, tolerability and acceptability of tDCS

Three repeated measures ANOVAs revealed no effect of stimulation type on blood pressure or pulse [all *p* ≥ .104]. An additional repeated measures ANOVA revealed a main effect of stimulation on discomfort ratings [*F*(2, 76) = 5.82, *p* = .004]. Simple effects analyses showed that both real conditions were rated as more uncomfortable than sham tDCS [AR/CL: *F*(1, 38) = 7.14, *p* = .011, *r* = 0.40, AL/CR: *F*(1, 38) = 10.05, *p* = .003, *r* = 0.46]. Nevertheless, all sessions were associated with low levels of discomfort (10cm VAS, AR/CL: *M* = 2.82, *SD* = 2.40, AL/CR: *M* = 2.88, *SD* = 2.23, sham: *M* = 1.72, *SD* = 1.54). Thirty-eight of 39 participants indicated that they would consider taking part in a therapeutic trial of tDCS (the remaining participant was unsure).

## 4. Discussion

This is the first study to investigate the effects of tDCS in BN. The results provide positive proof-of-principle for the clinical utility of bilateral tDCS applied to the DLPFC in this patient population. Specifically, AR/CL tDCS transiently reduced the severity of ED-related cognitions (indexed by the MEDCQ-R) when compared with AL/CR and sham tDCS. In addition, both AR/CL and AL/CR suppressed the urge to binge-eat and increased the level of self-regulatory control exercised during a TD task. Compared to sham stimulation, mood (assessed with the POMS) improved after AR/CL but not AL/CR tDCS. Lastly, the three tDCS sessions exerted equivalent effects on the wanting and liking of food and on bulimic behaviours during the 24-hour follow-up period.

The decrease in symptoms of BN is consistent with emerging evidence demonstrating that modulation of the DLPFC with NIBS can induce therapeutic effects in EDs [26, 32, 33, 46, 52]. Though only modest improvements were recorded and no effects on actual bulimic behaviours were observed, cortical excitability changes generated by a single session of tDCS are slight (~20%) and appear to diminish approximately 1–2 hours post-stimulation [53, 54]. Conversely, tDCS interventions comprising multiple sessions have been shown to produce consolidative and cumulative excitatory effects [54], and to elicit long-lasting clinical gains in several psychiatric disorders [31], including anorexia nervosa [33]. The results of this study therefore provide a strong rationale for conducting a tDCS treatment trial in BN.

Our finding that active but not sham tDCS reduced TD behaviour contributes to research showing that this presumed stable personality trait can be altered acutely by experimental manipulation [46, 55, 56], and suggests that the absence of change previously documented by our group [29] might be attributable to differences in the TD task/measurement of discounting. It also supports a key role for the DLPFC in self-regulatory control, and provides some insight into the neurocognitive mechanisms through which tDCS might exert its therapeutic effects. Further mechanistic inferences can be drawn from our finding that real versus sham tDCS reduced total mood disturbance (though only at trend level), which corresponds to data from the depression literature [57]. As negative affect is generally elevated prior to binge-eating and purging [58], improvements in mood are likely to facilitate improvements in clinical symptoms.

In this study, real versus sham stimulation had no impact on the wanting or liking of a selection of sweet and savoury high- and low-calorie foods, which contradicts the anti-craving effects of tDCS reported previously [29, 59–61]. Nevertheless, these prior studies were conducted in healthy individuals, whose cravings may have been less intense and more modifiable. It may also be that our method of assessment lacked sensitivity, since the craving index in our earlier study [29] incorporated ratings of the sensory properties of the foods presented (i.e., smell, taste, and appearance), and cravings are known to represent desire for particular sensory stimulation [62]. Alternatively, motivational tendencies towards food might be best captured with tasks assessing implicit as opposed to explicit wanting, such as those measuring reaction time [47] or saccadic eye movement [60].

The polarity effect identified in the present study—which favoured AR/CL over AL/CR tDCS—is in agreement with data from individuals with frequent food cravings [60], and suggests that ED cognitions and mood disturbance may be hemispherically lateralised in BN. Indeed, cortical asymmetry has been reported in relation to a number of ED symptoms; for example, overeating and decision-making impairments have been associated with reduced function in the right prefrontal cortex (PFC) [63], while disinhibition and appetitive responsivity have been linked to greater left-sided prefrontal activation [64]. These findings support the therapeutic potential of anodal (excitatory) and cathodal (inhibitory) modulation of the right and left DLPFC, respectively, but do not explain why AL/CR tDCS (and excitatory rTMS to the left DLPFC [27]) too produces beneficial effects. It is also unclear why AL/CR stimulation failed to improve mood in this study, when this tDCS montage has been successfully used to treat major depression [57]. Additional neuroimaging data may foster a greater understanding of brain laterality in BN.

This study has several limitations. Firstly, by chance, there was a trend for pre-tDCS urge to binge-eat scores to differ across the conditions: slightly lower values were obtained before sham tDCS than before AR/CL and AL/CR tDCS (Fig 3), allowing less scope for improvement in the sham session. Similarly, pre-tDCS MEDCQ-R scores appeared to be highest before AR/CL tDCS (Fig 4), although this difference did not approach significance. Secondly, due to the high levels of psychiatric comorbidity associated with BN [65], we did not exclude individuals with co-occurring mental disorders. While it seems unlikely, it is not possible to state whether an effect of tDCS on comorbid psychiatric symptoms contributed to the findings [66]. Thirdly, we included both left- and right-handed participants, and handedness influences the effects of rTMS in BN [67]. Fourthly, although the researcher administering the experimental measures was blind to the stimulation condition, we cannot rule out the possibility that participants were influenced by interaction with the unblinded tDCS technician. Lastly, data on ED symptoms immediately after stimulation were gathered using self-report psychological measures. This limits the clinical applicability of our findings since the principal goal of BN treatments is normalisation of eating behaviour.

## 5. Conclusions

The current research provides preliminary evidence that bilateral tDCS to the DLPFC has the potential to induce therapeutic effects in BN, at least temporarily. It also elucidates possible mechanisms of action and informs the design of future trials, particularly in relation to electrode montage selection. While only modest conclusions can be drawn regarding the clinical utility of tDCS in BN, our findings offer support and justification for studies involving multi-session protocols.

## Supporting Information

S1 Appendix Full trial protocol. (DOCX)

S2 Appendix CONSORT checklist. (DOC)

S3 Appendix Raw data. (SAV)
