# Supplementary material for: Single-Session Transcranial Direct Current Stimulation Temporarily Improves Symptoms, Mood, and Self-Regulatory Control in Bulimia Nervosa: A Randomised Controlled Trial
Source: PLoS One. 2017 Jan 25;12(1):e0167606. doi: 10.1371/journal.pone.0167606 (PMC5266208; doi:10.1371/journal.pone.0167606)
Supplement: S1 Appendix — (DOCX) [file pone.0167606.s001.docx]

**Transcranial direct current stimulation in anorexia nervosa and bulimia nervosa**

Aims and objectives

The primary aim of this study is to establish whether a single session of sham-controlled transcranial direct current stimulation (tDCS) applied to the dorsolateral prefrontal cortex (DLPFC) will reduce eating disorder (ED)-related thoughts and behaviours in individuals with anorexia nervosa (AN) and in individuals with bulimia nervosa (BN). The secondary aim is to investigate the effects of tDCS on self-regulation and emotional state in these patients. In addition, this study will examine whether the effects of tDCS are moderated by the polarity of the electrodes.

Scientific background

AN and BN are serious psychiatric disorders associated with substantial morbidity and mortality. Although the treatment experience of individuals with these EDs is highly variable, overall recovery rates are relatively low; thus highlighting the need for novel strategies.

Over the past decade much progress has been made in understanding the neural underpinnings of EDs. Evidence indicates that the control of eating is associated with neural networks in the prefrontal cortex that are also involved in more general self-regulatory processes. Research shows that individuals with AN and BN typically possess maladaptive self-regulation skills on a global scale; therefore a deficiency in the prefrontal cortical networks might contribute to the aetiology of these EDs. Indeed, functional magnetic resonance imaging (fMRI) studies have revealed altered neuronal activity in the brains of ED patients, compared to healthy controls, in several anatomical regions including the frontal lobes. Given the unsatisfactory response rates of current psycho- and pharmacotherapies, these emerging neural models of EDs provide a strong rationale for the use of targeted brain-directed interventions, especially in severe and enduring ED cases.

Non-invasive brain stimulation (NIBS) procedures – namely repetitive transcranial magnetic stimulation (rTMS) and tDCS – can be used to directly manipulate neuronal excitability with minimal side effects, and have demonstrated therapeutic potential in a wide range of neuropsychiatric disorders. Moreover, rTMS has recently been approved by the US Food and Drug Administration (FDA) agency as a second-line treatment for major depressive disorder (MDD), and has shown particular promise in treatment-resistant patients. This provides further reasoning for the use of NIBS in the treatment of EDs since many biological similarities exist between AN, BN, and MDD.

So far several studies have shown that NIBS can effectively reduce ED symptoms; for example, our group found that one session of high-frequency rTMS delivered to the left DLPFC lowered cue-induced food cravings in people with a bulimic disorder, and reduced levels of feeling full, fat, and anxious in patients with AN (Van den Eynde et al., 2010, 2013). In addition, tDCS has been shown to reduce food cravings in healthy participants (Fregni et al., 2008; Goldman et al., 2011; Kekic et al., in preparation) and the desire to eat in overweight and obese participants (Montenegro et al. 2012). Our group recently reviewed the effects of neuromodulation on eating and body weight systematically and concluded that NIBS tools have the potential to alter disordered eating behaviours (McClelland et al., 2013a). Although the effects recorded in most studies were only temporary, it is possible that NIBS interventions delivered over longer periods of time could serve as effective adjunctive strategies for the treatment of EDs. Indeed, the administration of multiple doses of rTMS has improved symptoms in AN patients (McClelland et al., 2013b) and induced complete remission in BN patients (Hausmann et al., 2004; Downar et al., 2012).

While rTMS and tDCS tend to yield similar results, tDCS is the more practical of the two techniques; it is simpler, less expensive, and raises fewer safety concerns than rTMS. In addition, the ability of tDCS to simultaneously excite and inhibit cortical neurons seems ideal for rectifying the inter-hemispheric imbalance that is seen in the brains of ED patients (Hecht et al., 2010). Despite these advantages, tDCS is yet to be trialled in individuals with AN or BN. The proposed investigation therefore aims primarily to establish whether a single session of prefrontal cortex tDCS will reduce eating disorder-related thoughts and behaviours in individuals with AN and in individuals with BN.

Methodology

*Participants*

72 adults will be recruited – 36 with a DSM-V diagnosis of AN or eating disorder not otherwise specified-anorexia type (EDNOS-AN) and 36 with a DSM-V diagnosis of BN or eating disorder not

otherwise specified-bulimia type (EDNOS-BN). Using a paired samples t-test to detect differences between means, and assuming an α level of 5% using a two-sided test, this sample size will have 80% power to detect a medium effect size (0.5). This takes into account an estimated 5% dropout.

*Design*

A crossover design will be conducted in which all participants will receive anode right/cathode left tDCS, anode left/cathode right tDCS, and sham tDCS. A 48-hour interval will be used between sessions to avoid any carryover effects due to stimulation. The order of stimulation will be randomised and counterbalanced across participants using STATA® – software the random allocation sequence will be created by a third party and given to the experimenter in opaque sealed envelopes. For the AN group, randomisation will be stratified according to AN subtype (restrictive vs. binge/purge). The researcher administering the tDCS will be unblinded to the type of stimulation being administered; however, the researcher administering the experimental measures and the participant will both be blinded.

*Procedure*

1. Participants will be screened by phone for inclusion/exclusion criteria (including the completion of a tDCS safety screen). The eating disorders module of the Structured Clinical Interview for DSM Axis I Disorders (SCID-I) will also be conducted. Eligible participants will be scheduled in for testing and non-eligible participants will be informed and thanked for their time.

2. On day one participants will give written consent and complete two baseline assessments – the Eating Disorder Examination Questionnaire (EDEQ) and the 21-item Depression Anxiety and Stress Scale (DASS-21). Demographic information will also be obtained.

3. A number of experimental procedures will then take place in the following order:

(a) Hormonal stress response: a saliva sample will be taken so that levels of cortisol can be measured and the stress associated with the procedure can be recorded.

(b) Visual analogue scales (VASs): measuring emotional state, level of preoccupation with food and body-image, and attitude towards food intake.

(c) Computerised Delay Discounting (DD) task: to measure self-regulation, over 100 trials participants will choose between a smaller amount of money available immediately and a larger amount available after a delay.

(d) Food Challenge Task (FCT): participants will watch a short film (< 5 minutes) showing highly palatable foods (crisps, nuts, biscuits, and chocolates) which will also be present on a tray in front of them. Participants will rate on VASs the appearance, smell, and taste of the foods as well as their urge to eat each one.

(e) Repeat visual analogue scales (VASs): measuring emotional state, level of preoccupation with food and body-image, and attitude towards food intake.

(f) Blood pressure and pulse measurements.

4. Next, participants will receive a 20-minute tDCS session (anode right/cathode left, anode left/cathode right, or sham) administered using a neuroConn® device. Half way through this session (after 10 minutes) blood pressure and pulse measurements will be taken again.

5. After the tDCS session, the experimental procedures listed above will be repeated in the same order.

6. Participants will then rate their level of discomfort during the tDCS procedure on a VAS.

7. 24 hours later participants will be emailed a short questionnaire about ED-related thoughts/behaviours experienced/engaged in during the 24-hour period following the laboratory session.

8. 48 hours after day one participants will return for day two. After another 48-hour interval, participants will return for day three. The same protocol will be repeated on each laboratory visit; however, participants will only complete the baseline questionnaires on day one. Furthermore, they will receive a different type of stimulation on each day (anode right/cathode left vs. anode left/cathode right vs. sham).

9. Upon completion of the three laboratory sessions and the three follow-up questionnaires, participants will be thanked for their involvement in the study and reimbursed for their time, efforts and travel (£50). The success of the blinding procedure will also be evaluated and participants will be asked whether they would be happy to take part in a therapeutic trial of tDCS if this were available.

See Table 1 for a diagram of the procedure.

*Data analysis*

Data will be analysed using SPSS®. Assuming that data are normally distributed, the effects of tDCS will be evaluated using two-way 3 (stimulation: anode right/cathode left vs. anode left/cathode right vs. sham) x 2 (timepoint: pre-tDCS vs. Post-tDCS) repeated measures ANOVAs, whereby a significant stimulation x timepoint interaction would indicate that the three tDCS sessions had differing effects on pre-tDCS measures. Post-hoc tests will be conducted to determine which difference(s) between conditions gave rise to the overall result.

***Table 1.*** tDCS in AN and BN study procedure

References

Downar, J., Sankar, A., Giacobbe, P., Woodside, B. & Colton, P. (2012). Unanticipated rapid remission of refractory bulimia nervosa, during high-dose repetitive transcranial magnetic stimulation of the dorsomedial prefrontal cortex: a case report. *Frontiers in Psychiatry, 3,* 1-5.

Fregni, F., Fernanda, O., Pedrosa, W., Fecteau, S., Tome, F., Nitsche, M…& Boggio, P. (2008a). Transcranial direct current stimulation of the prefrontal cortex modulates the desire for specific foods. *Appetite, 51,* 34-41.

Goldman, R.L., Borckardt, J.J., Frohman, H.A., O’Neil, P.M., Madan, A., Campbell, L.K…& George, M.S. (2011). Prefrontal cortex transcranial direct current stimulation (tDCS) temporarily reduces food cravings and increases the self-reported ability to resist food in adults with frequent food craving. *Appetite, 56,* 741-746.

Hausmann, A., Mangweth, B., Walpoth, M., Hoertnagel, C., Kramer-Reinstadler, K., Rupp, C.I. & Hinterhuber, H. (2004). Repetitive transcranial magnetic stimulation (rTMS) in the double-blind treatment of a depressed patient suffering from bulimia nervosa: a case report. *International Journal of Neuropsychopharmacology, 7,* 371-373.

Hecht, D. (2010). Transcranial direct current stimulation in the treatment of anorexia. *Medical Hypotheses, 74,* 1044-1047.

McClelland, J., Bozhilova, N., Campbell, I. & Schmidt, U. (2013a). A systematic review of the effects of neuromodulation on eating and body weight: evidence from human and animal studies. *European Eating Disorders Review, 21,* 436-455.

McClelland, J., Bozhilova, N., Nestler, S., Campbell, I., Jacob, Johnson-Sabine, E. & Schmidt, U. (2013). Improvements in symptoms following neuronavigated repetitive transcranial magnetic stimulation (rTMS) in severe and enduring anorexia nervosa: findings from two case studies. *European Eating Disorders Review, 21,* 500-506.

Montenegro, R.A., Okano, A.H., Cunha, F.A., Gurgel, J.L., Fontes, E.B. & Farinatti, P.T. (2012). Prefrontal cortex transcranial direct current stimulation associated with aerobic exercise change aspects of appetite sensation in overweight adults. *Appetite, 58,* 333-338.

Van den Eynde, F., Claudino, A.M., Mogg, A., Horrell, L., Stahl, D., Ribeiro, W…& Schmidt, U. (2010). Repetitive transcranial magnetic stimulation reduces cue-induced food craving in bulimic disorders. *Biological Psychiatry, 67,* 793-795.

Van den Eynde, F., Guillaume, S., Broadbent, H., Campbell, I. & Schmidt, U. (2013). Repetitive transcranial magnetic stimulation in anorexia nervosa: a pilot study. *European Psychiatry, 28,* 98-101.
